# Postoperative Atrial Fibrillation: Evaluation of its Economic Impact on the Costs of Cardiac Surgery

**DOI:** 10.21470/1678-9741-2018-0218

**Published:** 2019

**Authors:** Edgar Hernández-Leiva, Paula Alvarado, Rodolfo José Dennis

**Affiliations:** 1 Department of Cardiology, Fundación Cardioinfantil - Instituto de Cardiología, Bogotá DC, Colombia.; 2 Internal Medicine, Fundación Cardioinfantil - Instituto de Cardiología, Bogotá DC, Colombia.

**Keywords:** Atrial Fibrillation, Cardiac Surgery, Postoperative Care, Cost-of-Disease

## Abstract

**Objective:**

The objective of this study was to calculate the direct costs of postoperative atrial fibrillation (POAF) in a high-complexity cardiovascular hospital.

**Methods:**

We performed a cost analysis with a pairwise-matched design. Twenty-two patients with POAF and 22 patients without this complication were included. Pair-matching was performed (1:1) based on the following criteria: identical type of surgery, similar EuroSCORE II values, and absence of any other postoperative complication.

**Results:**

The total hospital cost was significantly higher in the POAF group than in the non-POAF group (US$ 10,880 [± 2,688] *vs*. US$ 8,856 [± 1,782], respectively, for each patient; *P*=0.005). This difference was attributable to postoperative costs (US$ 3,103 [± 1,552] *vs*. US$ 1,238 [± 429]; *P*=0.0001) for patients with or without POAF, respectively. The median postoperative lengths of stay were 9 (range 5-17) and 5 (3-9) days for patients with and without POAF (*P*=0.032), respectively. Preoperatively, no differences were found in the EuroSCORE II values (median 1.7 *vs*. 1.6, respectively; *P*=0.91) or direct costs (US$ 1,127 *vs*. US$ 1,063, respectively; *P*=0.56) between POAF and non-POAF groups.

**Conclusion:**

POAF generates a high economic burden in the overall costs of cardiac surgery, and our results reveal the differential contribution of each of the evaluated factors. This information, which was previously unavailable in this setting, is essential for the development of more effective prevention strategies.

**Table:** 

Abbreviations, acronyms & symbols	
AF	= Atrial fibrillation
CABG	= Coronary artery bypass grafting
CD	= Cost-of-disease
CI	= Confidence interval
CVICU	= Cardiovascular intensive care unit
OR	= Odds ratio
POAF	= Postoperative atrial fibrillation
SD	= Standard deviation
TAVR	= Transcatheter aortic valve replacement

## INTRODUCTION

Postoperative atrial fibrillation (POAF) is the most common complication of cardiac surgeries, with reported incidence rates between 10% and 65%, depending on the cohort under study, definition, and detection method used^[1-2]^. This complication is strongly associated with advanced age^[3]^; therefore, the progressive increase in the average age of cardiac surgery patients in recent years requires a greater understanding of the impact of POAF on clinical outcomes, the hospital resources consumed, and the costs of medical care. Previous studies have shown that POAF continues to be an important determinant of the duration of postoperative stay, use of resources, and incidence of readmission^[4-7]^. However, the economic impact on the costs of cardiac surgery has not been evaluated in the current setting. This study seeks primarily to determine the direct medical costs of POAF and their source, as well as evaluate if these results vary according to the type of surgery.

## METHODS

This study was conducted in a 500-bed private tertiary care clinic with a cardiovascular focus. All adult patients who underwent elective cardiac surgery within a period of 23 months were included.

### Study Design

This study is a cost-of-disease (CD) analysis with a prevalent approach and it was planned as a pairwise-matched study^[8]^, by matching patients with and without POAF (1:1) concurrently. For identification purposes, patients were classified into one of two groups according to whether they presented POAF: the POAF (+) group and the POAF (-) group. This type of study design was considered necessary because costs must be calculated against a counterfactual scenario in which the population would have a hypothetical alternative occurrence of POAF but would be identical in all other aspects^[9]^.

POAF was identified through continuous postoperative monitoring, which is already conducted in the cardiovascular intensive care unit (CVICU), and later through telemetry during the hospital stay. POAF was defined as the occurrence of any episode of atrial fibrillation (AF) or flutter with a duration of 30 seconds or longer (collectively called AF for this analysis) occurring postoperatively until discharge and documented in an electrocardiographic trace^[10]^.

To study the independent effect of the variable of interest (POAF) on costs, the influence of certain variables, such as surgery type, comorbidities, and the absence of other postoperative complications, was controlled; these variables were the matching criteria.

Of the two epidemiological perspectives that correspond to the interpretation of CD studies, the prevalence-based approach was considered the most appropriate to evaluate the actual economic burden of this health problem, given that it is the main method chosen for the assessment of short-term acute conditions^[11]^.

### Patients

All adult patients who underwent elective cardiac surgery comprising one of these three procedures were included:

- On-pump coronary bypass surgery.
- Aortic valve replacement.
- Mitral valve replacement or repair.

Patients were evaluated daily to record the occurrence of POAF. Those with a history of any preoperative supraventricular arrhythmia were excluded. Additionally, those subjected to percutaneous surgical procedures [transcatheter aortic valve replacement (TAVR), aortic endoprosthesis, and other procedures)], minimally invasive procedures, hybrid procedures, heart transplant, or surgeries in which double procedures were performed were also excluded. These exclusions made it challenging to appropriately match patients with certain procedures, which resulted in uncertainty regarding the generalization of the results obtained.

Given that statistical data of all patients during the postoperative period after cardiac surgery are systematically recorded in the cardiovascular intensive care unit (CVICU), POAF (-) patients were chosen as the first patients, after POAF (+) patients, when they met the defined pairing criteria in the following order:

1. No episode of POAF.
2. Identical type of surgery.
3. Similar EuroSCORE II values (as a global criterion of preoperative morbidity and intraoperative risk), within a range of ± 1.5% with respect to the calculated point estimate
4. Absence of any other postoperative complication (*).

(*) Postoperative complications included clinical or laboratory procedures that, in the opinion of the researchers, could have a significant impact on costs (applies to both groups).

### Study Objectives

- To define the direct medical costs of POAF.
- To determine the source of the costs (length of stay, medications, laboratory procedures, diagnostic images, medical-surgical supplies, physical therapy, and rehabilitation).
- To evaluate if these results vary according to the type of surgery (sensitivity analysis).

### Outcomes

The main outcomes of interest evaluated were the direct medical costs (intrahospital or 30 days postoperative costs, whichever occurred first), which are described in Table 1. The study was not designed to analyze associations among clinical outcomes. However, the comorbidities that comprise the EuroSCORE II, the events of operative morbidity and mortality, and durations of stay in the hospital and CVICU were recorded.

**Table 1:** Source of postoperative costs.

	POAF (+) Monetary value US$ (mean and SD)	POAF (-) Monetary value US$ (mean and SD)	*P* value
Total cost	3,103 (± 1.552)	1,238 (± 429)	0.0001
Hospital stay	1.451 (± 691)	553 (± 223)	0.0001
Medicines	568 (± 354)	178 (± 113)	0.0001
Laboratory procedures	365 (± 288)	166 (± 68)	0.02
Diagnostic imaging	240 (± 82)	136 (± 76)	0.008
Medical-surgical supplies	259 (± 291)	74 (± 42)	0.0001
Physical therapy and rehabilitation	174 (± 130)	117 (± 66)	0.38

Not all variables show a normal distribution; however, for clarity, all data are shown as the means and standard deviations (SD). All costs are expressed in US dollars using the exchange rate for November 2017. POAF=postoperative atrial fibrillation

The lengths of hospital and CVICU stays were quantified for each pair of patients group, POAF (+) group *vs*. POAF (-) group, and the incremental prolongation of the stay was calculated. The perspective of the third-party payer was chosen for the cost analysis.

All the resources used were documented from the admission to the CVICU until the hospital discharge or 30 postoperative days. The use of resources specifically refers to DIRECT MEDICAL COSTS for the third-party payer, including the costs of the stay, medications, supplies, tests, and procedures. The unit costs were obtained from the billing system to reflect the hospital compensation for each item. All financial information was adjusted to 2017 values considering current rates updated at the time of analysis. The costs were obtained in Colombian pesos and converted into US dollars (United States of America), based on the exchange rate at the end of the analysis (November 2017).

### Ethical Aspects

The research proposal was approved by the Institutional Research Ethics Committee. The study was exempted from informed consent because it was an anonymous analysis of data recorded in medical records or billing files and did not affect the study plans or the patient's treatment.

### Statistical Analysis

The analyses were performed with STATA 13.0 software (StataCorp LLC. TX 77845, USA). Categorical variables are expressed as percentages, and continuous variables are expressed as means and standard deviations (SD) or medians [25^th^ and 75^th^ percentiles], according to their global distribution.

The study was conducted according to the econometric approach described by Changik Jo in a CD review^[12]^, which compared the differences in mean costs supported by each of the two cohorts to determine the incremental difference attributable to POAF. The two groups were made comparable by one-to-one pairing to control potentially confounding variables.

The first step in the statistical analysis was to define the characteristics of distribution and variance of the variables under study. To compare the differences in costs found between the groups, Student's or Welch's t-test was used depending on the variance. For variables that did not meet the normality criteria, the nonparametric Wilcoxon test was used. The resulting analyses were considered statistically significant if the *P* values were fewer than 0.05.

### Calculation of the Sample Size

A priori, it was considered that the prolongation of the hospital stay would be the most important factor in the increase of costs. According to the mean value and standard deviation found in a previous global sample of patients and using the formula for difference between means described by Dennis^[13]^, with a power of 0.8 and an alpha level of 0.05 for a one-tailed test, a sample size of 16 patients in each group was calculated. Accounting for the costs of other variables and after adding an additional 10% for eventual losses of data necessary for the analyses, a final sample of 44 patients was obtained (22 in each group). The researchers considered that alternative methods of calculating the sample size could not be applied to this study design.

## RESULTS

**Fig. 1:**
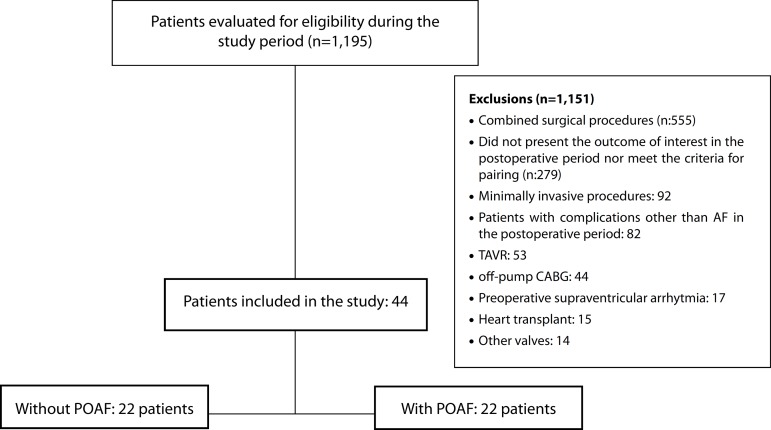
Flowchart of the progress through the study phases. AF=atrial fibrillation; CABG=coronary artery bypass grafting; POAF=postoperative atrial fibrillation; TAVR=transcatheter aortic valve replacement

A total of 1,195 patients underwent cardiac surgical procedures during the 21-month study period; see the flow chart in Figure 1.

### Characteristics of the Patients and Procedures

The study population consisted of 44 patients with an average age of 66.2 (± 8.4) years, 17 of whom were women (38.6%).

Table 2 shows that no significant difference was found between the groups with respect to their baseline risk of postoperative morbidity and mortality (estimated by the EuroSCORE II). Although the patients' age is older in the POAF (+) group, this difference was not significant. The types of surgery and number of patients are described in Table 3.

**Table 2:** Baseline characteristics.

	POAF (+)	POAF (-)	*P* value
Age, mean (±SD)	67.9 (± 8.5)	64.4 (± 8.2)	0.08
Median EuroSCORE II (range)	1.73 (0.5-7.7)	1.65 (0.65-9)	0.91

The distribution by groups of each of the variables that composed the EuroSCORE II (age, gender, renal function, extracardiac arteriopathy, impaired mobility, previous cardiac surgery, chronic lung disease, active endocarditis, critical preoperative state, insulin-dependent diabetes mellitus, functional class, angina at rest, left ventricular function, recent myocardial infarction, pulmonary hypertension, emergency surgery, risk of intervention, and surgery on the thoracic aorta). POAF=postoperative atrial fibrillation; SD=standard deviation

**Table 3:** Type of surgery.

	POAF (+) Number of patients	POAF (-) Number of patients
Myocardial revascularization	11	11
Aortic valve replacement	8	8
Mitral valve replacement	1	1
Myocardial revascularization + mitral valve replacement	1	1
Myocardial revascularization + mitral valve repair	1	1

POAF=postoperative atrial fibrillation

### Time of Stay and Costs

Table 4 shows why the total and postoperative stay times were significantly prolonged in the subgroup with POAF (in both the CVICU and the general hospital ward). Table 5 shows that the total costs were significantly higher in the POAF group, with an increase of approximately US$ 2,000 for each patient presenting this outcome. It should be noted that the preoperative costs are not different.

**Table 4:** Clinical outcomes.

	POAF (+) group	POAF (-) group	*P* value
Total hospital stay in days (mean [SD])	13.1 (± 4.5)	9.5 (± 4.8)	0.008
Postoperative CVICU stay in days (median [SD])	3.5 (1-11)	1 (1-3)	0.00001
Postoperative stay on the hospitalization floor in days (mean [SD])	4.9 (± 1.7)	3.8 (± 1.3)	0.012
Total postoperative stay in days (median [range])	9 (5-17)	5 (3-9)	0.032
Number of patients readmitted to CVICU (median)	12	0	0.0001

CVICU=cardiovascular intensive care unit; POAF=postoperative atrial fibrillation; SD=standard deviation

**Table 5:** Costs.

	POAF (+) US$	POAF (-) US$	*P* value
Preoperative cost, median (range)	1,127 (0-3,796)	1,063 (0-3,534)	0.56
Postoperative cost, median (range)	2,596 (1,324-5,112)	1,225 (671-2,318)	0.00001
Total cost, mean (SD)	10,880 (± 2,688)	8,856 (± 1,782)	0.005

All costs are expressed in US dollars using the exchange rate for November 2017. POAF=postoperative atrial fibrillation; SD=standard deviation

### Origin of Cost Overruns

**Fig. 2:**
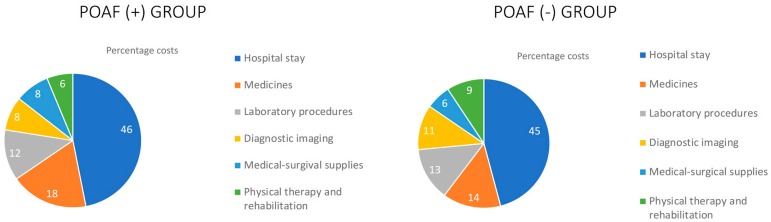
Origin of postoperative costs. POAF=postoperative atrial fibrillation

Postoperative costs account for most of the differences in the total cost found between the two groups. For purposes of the analysis, these postoperative costs were discriminated into six groups according to their origin. Table 1 and Figure 2 show that the main source of postoperative costs corresponds to the hospital stay, but significant increases are also present in the number of medications, laboratory procedures, diagnostic aids, and medical-surgical supplies.

### Sensitivity Analysis by Type of Surgery

The costs or their origin are not affected by the type of surgery. See Tables 6 and 7.

**Table 6:** Total postoperative cost and hospital stay in the subgroup of myocardial revascularization patients.

	POAF (+) Monetary value US$ (mean and SD)	POAF (-) Monetary value US$ (mean and SD)	*P *value
Total cost	2,684 (± 1,163)	1,292 (± 372)	0.0003
Hospital stay	1,304 (± 627)	571 (± 233)	0.001

All costs are expressed in US dollars using the exchange rate for November 2017. POAF=postoperative atrial fibrillation; SD=standard deviation

**Table 7:** Total postoperative cost and hospital stay in the subgroup of patients with other surgeries.

	POAF (+) Monetary value US$ (mean and SD)	POAF (-) Monetary value US$ (mean and SD)	*P* value
Total cost	3,523 (± 1.354)	1,184 (± 472)	0.000006
Hospital stay	1,598 (± 720)	535 (± 212)	0.0001

All costs are expressed in US dollars using the exchange rate for November 2017. POAF=postoperative atrial fibrillation; SD=standard deviation

## DISCUSSION

This is the first report on the impact of the costs of POAF after cardiac surgery in Latin America. With the data obtained, we calculated an excess of total in-hospital cost of approximately US$ 2,000 per patient. In absolute terms, the main component of this cost is the increase in the length of hospital stay; however, the findings likely underestimate the true cost of POAF because other potential costs exist. POAF can be recurrent during the first weeks of the postoperative period^[14]^, which implies that patients may have consulted medical services for this arrhythmia and were readmitted to other hospitals. Additionally, POAF has been associated with several postoperative complications, including deep sternal wound infection^[4]^, cerebrovascular events^[15]^, gastrointestinal complications^[4,16]^, pneumonia^[4]^, and renal failure^[4,17]^; if causal relationships with these complications were considered, the attributable costs would be enormous.

Comparisons with the results of other studies are complicated by the local nature of the costs and their variation over time. A study by Rostagno et al.^[18]^ evaluated the economic burden of the POAF and found an extra cost of US$ 2,593 (original data published in euros, in 2010), of which approximately 50% was due to the excess cost of hospital stay. In a publication by Hravnak et al.^[19]^ on patients undergoing coronary revascularization, those who developed POAF had in-hospital costs that were US$ 6,356 higher than their counterparts without POAF (2002). Aranky et al.^[20]^, in a regression analysis adjusting for comorbidities and other postoperative complications, reported that the number of additional days of hospitalization attributable to POAF was 4.9, which corresponded to an extra US$ 10,055 to US$ 11,500 of hospital charges per patient (1996). In their work, Doering LV et al.^[21]^ found that the occurrence of postoperative arrhythmia was a variable that was independently associated with higher costs, with an odds ratio (OR) of 3.5 (95% of confidence interval [CI] 1.1-10.6).

In a similar study conducted by Mauldin et al.^[22]^, significant cardiac arrhythmia was demonstrated in 25% of patients; the increase in the cost associated with arrhythmia compared to patients without complications was approximately US$ 6,000 per patient. Similar results have been reported by other authors, with additional costs attributable to POAF ranging from US$ 5,000 to US$ 12,000 per patient^[18,23]^.

To this date, studies have described the cost perspective of POAF on an individual basis for each patient, but it is likely more important to analyze its overall impact on a cardiac surgery program. Taylor et al.^[23]^ reported that in the postoperative period of a cardiac surgery, complications that may occur, when evaluated individually, generate more costs than POAF; however, because POAF is the most frequent complication (10-65% of all patients), its accumulated cost will exceed that of any other factor. These authors examined the economic consequences of postoperative complications associated after myocardial revascularization surgery. Respiratory failure and wound infection of the sternum were the most costly complications, but they occurred in only 3% and 0.4% of patients, respectively, while POAF was less expensive, but it was the most common complication, occurring in 20% of patients. Assuming a similar incidence of POAF in a cardiovascular hospital with a volume similar to ours (approximately 600 adult surgeries each year), the overcosts could generate an excess of US$ 600,000 annually.

The average cost overruns of POAF that we found in our study are lower than those described in the literature; however, this is not only attributable to the well-documented difference in the costs of cardiac surgery in industrialized and developing countries^[24]^. A second important factor is that to fulfill the objectives of the study, we have controlled the influence of the main comorbidities that usually accompany POAF and the different incidences of POAF according to the type of surgery by carefully pairing the subjects by the EuroSCORE values and requiring the same type of surgery for each pair to be compared. Few reports exist in the literature that have excluded patients who presented any other complication prior or subsequent to the occurrence of POAF; this measure allowed the actual and isolated costs to be refined, rather than evaluating a conglomerate of conditions that are associated with the occurrence of this postoperative complication. Finally, the increased in-hospital stay from 4 to 5 days in our series is consistent with that reported in older reports, of 7 to 10 days^[3,21]^; we believe that this change is mainly explained by the currently greater efficiency in the care processes.

The demonstration in our study that POAF is associated with an increase of more than US$ 2,000 in total costs of care, US$ 900 in the in-hospital stay, approximately US$ 400 in medications, US$ 300 in diagnostic tests and medical-surgical supplies, and US$ 200 in laboratory procedures, is currently of high relevance because great emphasis is placed on health care costs. These data also highlight an important question related to the adherence to management guidelines because although perioperative beta blockers are indicated as class I in POAF prevention^[25]^, our database shows that a proportion as high as 40% of patients referred for cardiac surgery do not receive this medication.

One aspect to be emphasized in this research is the pairing criteria, which were aimed at excluding confounding factors that could influence costs and therapeutic practices. In the literature, emphasis is placed on the methodology used in cost analysis due to the risk of biasing the results when not choosing the controls appropriately.

This study has limitations. Firstly, the data were obtained from a single center, which does not necessarily reflect the costs of all institutions, even in similar settings. Additionally, the analyses were limited to the short term, and clinical complications that frequently accompany POAF were not evaluated; however, the inclusion of associated conditions would introduce "noise" into the analyses, making it difficult to specify costs and to discriminate their origin.

## CONCLUSION

These results demonstrate that the occurrence of POAF is associated with a significant increase in the use of hospital resources and direct costs. Our data reflect the results of an institution focused on cardiovascular management and with a high patient volume, providing a frame of reference for current and future analyses related to the impact of this postoperative complication. The incidence of POAF is increasing, and potential strategies for the prevention of POAF and the appropriate selection of high-risk patients are still in development; therefore, cost information is essential for the design of cost-effectiveness studies.

**Table:** 

Authors' roles & responsibilities	
EHL	Directed all aspects of the study, including drafting the research proposal; presenting and defending the protocol to the Institution's Ethics and Research Committees, supervision of all the data collection of the study, review of the study's CRFs and data base, and drafting of the final report; approved the final manuscript
PA	Data collection and disseminated the objectives and scope of the research in the institution; approved the final manuscript
RJD	Advised the methodological aspects of the study, strengthening the epidemiological and statistical tools; contributed to the drafting of the study's written reports; approved the final manuscript
